# Blockage of Galectin-Receptor Interactions Attenuates Mouse Hepatic Pathology Induced by *Toxoplasma gondii* Infection

**DOI:** 10.3389/fimmu.2022.896744

**Published:** 2022-07-01

**Authors:** Jian He, Yongheng Hou, Fangli Lu

**Affiliations:** ^1^Department of Parasitology, Zhongshan School of Medicine, Sun Yat-sen University, Guangzhou, China; ^2^Department of Clinical Laboratory, The Seventh Affiliated Hospital, Sun Yat-sen University, Shenzhen, China; ^3^Key Laboratory of Tropical Disease Control of Ministry of Education, Sun Yat-sen University, Guangzhou, China

**Keywords:** *T. gondii*, C57BL/6 mice, Gal-9, CD137, hepatic pathology

## Abstract

*Toxoplasma gondii* (*T. gondii*), one of the most important Apicomplexan protozoa, causes toxoplasmosis in human throughout the world. Galectin (Gal)-9 triggers a series of immune events *via* binding to its receptors, including T cell immunoglobulin and mucin-containing molecule 3, CD137, CD44, and protein disulfide isomerase. To examine the regulatory role of galectin-receptor interactions in anti-toxoplasmic activities, C57BL/6 mice were infected with *T. gondii* RH strain and intraperitoneally injected with alpha (α)-lactose to block the interactions of galectins and their receptors. Heatmaps showed upregulated values for Gal-9 and CD137 in the livers of *T. gondii*-infected mice and *T. gondii*-infected mice treated with α-lactose. Compared with *T. gondii*-infected mice, *T. gondii*-infected mice treated with α-lactose showed significantly increased survival rate, decreased tissue parasite burden, attenuated liver histopathology, increased mRNA expression levels of CD137, IFNγ, IL-4, and IL-10 in the liver, and increased Gal-9 mRNA expression level in the spleen. Correlation analysis showed that significant positive correlations existed between the mRNA expression levels of Gal-9 and CD137, Gal-9 and IFNγ, as well as between CD137 and IFNγ in the liver and spleen of *T. gondii*-infected mice; between CD137 and IFNγ in the liver of *T. gondii*-infected mice treated with α-lactose. In addition, blockage of galectin-receptor interactions showed enhanced M2 macrophage polarization in the liver of *T. gondii*-infected mice. Our data indicate that Gal-9-CD137 interaction may play an important role in *T. gondii* proliferation and liver inflammation in mice during acute *T. gondii* infection, through regulating T cell and macrophage immune responses.

## Introduction

*Toxoplasma gondii* (*T. gondii*), a common and significant obligate protozoan parasite, is widely prevalent in humans and other animals on all continents (1). *T. gondii* infection can cause both congenital toxoplasmosis and postnatally acquired disease in immunocompromised patients (2), and its recrudescence can cause life-threatening disease in immunocompromised patients and recurrent ocular lesions in immunocompetent individuals (3). Toxoplasmic encephalitis is the most common cause of brain mass lesions in human immunodeficiency virus-infected patients (4). In addition, it has been reported a case of toxoplasmic hepatitis presenting with hepatomegaly in an immunocompetent patient (5). A study has confirmed the positive connection between toxoplasmosis and chronic liver diseases (6). The immune response against *T. gondii* infection is essential for parasite control and host survival, but it can also cause inflammation, tissue damage, and even death (7). Previous study has found that immune regulation may play an important role in modulating acute inflammatory pathogenesis and parasite control in the liver of mice infected with *T. gondii* (8).

Galectins are a class of β-galactoside-binding lectins that can bind to proteins by either *N*-linked or *O*-linked glycosylation (9). They are encoded by *Lgals* genes and differentially expressed by various immune cells, as well as a wide range of other cell types (10). Among the galectins, galectin (Gal)-9 is known for its immunomodulatory role in various microbial infections. It mediates host-pathogen interactions and regulates cell signaling *via* binding to its receptors (11). Gal-9 plays a crucial role in regulating effector T cells, and can transduce apoptosis of T helper (Th) 1 and Th17 cells by interacting with T cell immunoglobulin and mucin-containing molecule 3 (Tim-3), a transmembrane protein also known as CD366 (12, 13). In addition, Gal-9 can also bind to three other receptors, the co-stimulatory receptor CD137 (also known as 4-1BB and TNFRSF9) (14), CD44 (15), and protein disulfide isomerase (PDI) (16). CD137 is a co-stimulatory member of the tumor necrosis factor receptor (TNFR) superfamily distributed on T cell surface, to preferentially induce CD137 signaling, subsequent IFNγ production and Th1 response (14, 17). CD44 is a broadly distributed cell-adhesion molecule, which is important for leukocyte migration (18), as well as the stability and function of iTreg cells (15). PDI can specifically bind to Gal-9, and enhance T cell migration and viral entry (19). Our previous study has shown that the mRNA expression levels of Gal-9, Tim-3, and CD137 were increased in the cervical lymph nodes of both genetically *T. gondii*-susceptible C57BL/6 mice and *T. gondii*-resistant BALB/c mice, indicating that Gal-9 and its receptor interactions may play an important role in the immune regulation during acute ocular *T. gondii* infection (20).

Although *T. gondii* normally tends more to affect neurotropic and ocular organs, this parasite may also induce serious pathological changes in the liver (6), including hepatomegaly (5), granuloma hepatitis (21), cholestatic jaundice (22), and cirrhosis (23). Nonetheless, the immunopathological mechanisms those lead to the hepatic pathology in toxoplasmosis remain largely unknown. As an important immune protein highly expressed in the liver, Gal-9 presents a wide variety of biological functions involved in the maintenance of hepatic immune homeostasis and inflammation (24). However, it is not clear yet what roles of Gal-9 and its receptors play in the hepatic immunopathology during acute *T. gondii* infection. Therefore, the present study was designed to elucidate this question using *T. gondii*-infected murine models injected with alpha (α)-lactose to block galectin-receptor interactions and assess the consequent changes. Here we identified a critical role of Gal-9-CD137 interaction in the survival rate, parasite burden, liver pathology, and cytokine expressions in *T. gondii*-infected mice. These findings may provide the basis of developing galectin-receptor-targeted strategy for control of immunopathology during acute *T. gondii* infection.

## Materials and Methods

### Ethics Statement

Eight-week-old male C57BL/6 mice were purchased from the Animal Center of Sun Yat-sen University (Guangzhou, China), maintained in specific-pathogen-free environment, and had free access to a commercial basal diet and tap water *ad libtum*. Animals were provided with humane care and healthful conditions. All individuals who are involved in animal experiments received instruction in experimental methods and in the care, maintenance, and handling of mice; and all efforts were made to minimize animal suffering. Animals were sacrificed using CO_2_ asphyxiation and the appropriate organs were harvested. The protocol used in this study was approved by the Committee on the Ethics of Animal Experiments of Sun Yat-sen University [Permit Numbers: SCXK (Guangdong) 2016–0029].

### *T. gondii* and Experimental Infection

Tachyzoites of *T. gondii* RH strain were propagated by serial passage in human foreskin fibroblast-1 (HFF-1) cell (BNCC100406, BeNa Culture Collection, Beijing, China) monolayer grown in Dulbecco’s modified eagle’s medium (11995065, Gibco, Thermo Fisher Scientific Inc., Rockford, USA) supplemented with 10% (V/V) fetal bovine serum (10099141, Gibco, Thermo Fisher Scientific Inc.). After lysis of *T. gondii*-infected cells, tachyzoites were harvested by centrifugation at 2 000 × g for 5 min. The tachyzoites were enumerated using manual counting with a hemocytometer, and experimental *T. gondii*-infected mice were intraperitoneally (i.p.) injected with 1×10^2^ tachyzoites.

### α-Lactose Treatment

Galectins was blockaded by α-lactose to limit binding to their receptors as described previously (25, 26). A total of 42 C57BL/6 mice were divided into 4 groups: 11 mice were infected with *T. gondii* tachyzoites (*Tg-*infected mice); 11 mice were infected with *T. gondii* tachyzoites and injected i.p. with 27 mM of α-lactose (L8040-100, Sigma-Aldrich, St. Louis, MO, USA) solution in PBS (*Tg+*lact mice) twice daily starting from day 0 post infection (p.i.) until death of the mice; 10 mice were injected i.p. with α-lactose solution twice daily as α-lactose control (lact mice), while 10 mice were injected with equal volume of PBS twice daily as uninfected control (uninfected mice). Mortality was monitored daily. Another 16 mice were equally divided into four groups. They were sacrificed by CO_2_ asphyxiation on day 7 p.i., and the liver and spleen of each mouse were harvested for further analysis.

### Antibodies

Antibodies used for *in vitro* analysis include anti-CD86 mAb (GB13585) and anti-CD206 pAb (GB113497) (Wuhan Servicebio Biotechnologies Co., Ltd., Wuhan, China), anti-β-actin mAb (BM0627) (Wuhan Boster Biological Engineering Co., Ltd., Wuhan, China), anti-β-tubulin mAb (3G6) (Abbkine Inc., Wuhan, China), anti-38 kDa protein of *T. gondii* mAb (K97A) (Thermo Fisher Scientific Inc.), and anti-CD137 pAb (ab203391) (Abcam Plc., Waltham, USA).

### Measurement of mRNA Expression in Liver and Spleen Tissues by Using Quantitative Real-Time Polymerase Chain Reaction (qRT-PCR)

Total RNA was extracted from about 100 mg of liver or spleen from each mouse using the MiniBEST Universal RNA Extraction Kit (9767, TaKaRa Bio, Inc., Shiga, Japan) according to the manufacturer’s protocol. The quality of total RNA was analyzed by running 5 μl of each RNA sample on a 1.0% agarose gel stained with ethidium bromide. The quantity of total RNA was estimated by measuring the ratio of absorbance at 260 and 280 nm using a NanoDrop One spectrophotometer (NanoDrop Technologies, Inc., Wilmington, DE, USA). First-strand cDNA was constructed from 1.5 μg of total RNA with oligo (dT) as primers using the PrimeScript™ II 1st Strand cDNA Synthesis Kit (6210A, TaKaRa Bio Inc.). The cDNA products were stored at −80°C for use. To determine the mRNA levels of *T. gondii* major surface antigen 1 (SAG1), IFNγ, TNFα, IL-12p40, IL-4, IL-10, Gal-1, Gal-3, Gal-8, Gal-9, CD44, CD137, PDI, and Tim-3, qRT-PCR was performed using the SYBR^®^ Premix Ex Taq™ II Kit (Tli RNaseH Plus) (RR820A, TaKaRa Bio Inc.) according to the manufacturer’s instructions. The primer sequences are listed in **Table 1**. Briefly, a total of 20 μl reaction mixture contained 10 μl of SYBR^®^ Premix Ex Taq™ II (2×), 1 μl of each primer (8 μM), 6 μl of dH_2_O, and 2 μl of cDNA (0.2 μg/μl). Amplification was pre-denaturized for 30 s at 95°C, followed by 40 cycles of 5 s at 95°C and 30 s at 60°C using a CFX96 Touch^®^ Real-Time PCR Detection System (Bio-Rad Laboratories, Hercules, CA, USA). Specific mRNA expression levels were normalized to that of the housekeeping gene, GAPDH, and the results are expressed as fold change compared to uninfected mice.

**Table 1:** Primer sequences of genes used for quantitative real-time polymerase chain reaction assays.

Genes	Forward primers (5′→3′)	Reverse primers (5′→3′)	Accession No.
SAG1	CTGTCAAGTTGTCTGCGGAAGGAC	CGTTAGCGTGGCACCATTATCACTC	MK250980.1
IFNγ	GGAACTGGCAAAAGGATGGTGAC	GCTGGACCTGTGGGTTGTTGAC	NM_008337.4
TNFα	CCCTCACACTCAGATCATCTTCT	GCTACGACGTGGGCTACAG	AY423855.1
IL-12p40	CCTGGTTTGCCATCGTTTTG	TCAGAGTCTCGCCTCCTTTGTG	NM_001303244.1
IL-4	ACAGGAGAAGGGACGCCAT	GAAGCCCTACAGACGAGCTCA	NM_021283.2
IL-10	AGCCGGGAAGACAATAACTG	CATTTCCGATAAGGCTTGG	NM_010548.2
Gal-1	CGCCAGCAACCTGAATC	GTCCCATCTTCCTTGGTGTTA	NM_008495.2
Gal-3	GCTACTGGCCCCTTTGGT	CCAGGCAAGGGCATATCGTA	NM_001145953.1
Gal-8	GGGTGGTGGGTGGAACTG	GCCTTTGAGCCCCCAATATC	NM_001199043.1
Gal-9	GCAGGAGGGACTTCAGGTGA	GCCCCCACTGTCCGTTCT	NM_001159301.1
CD44	TGCAGGTATGGGTTCATAGAAGG	GTGTTGGACGTGACGAGGA	NM_001039150.1
CD137	CGTGCAGAACTCCTGTGATAAC	GTCCACCTATGCTGGAGAAGG	NM_001077508.1
PDI	CGCCTCCGATGTGTTGGAA	GAAGAACTCGACTAGCATGAGC	NM_007952.2
Tim-3	CCACGGAGAGAAATGGTTC	CATCAGCCCATGTGGAAAT	NM_134250.2
GAPDH	TTGATGGCAACAATCTCCAC	CGTCCCGTAGACAAAATGGT	BC023196.2

### Western Blot Assay

Hepatic tissues were treated with RIPA lysis buffer (P0013B, Beyotime, Haimen, China), and lysate was incubated on ice for 30 min. After centrifugation at 10, 000 × g for 5 min at 4°C, the protein concentration of each lysate was determined using a BCA Protein Assay Kit (P0010S, Beyotime). Total protein (20 μg) from each sample was boiled in SDS-PAGE loading buffer under reducing conditions, run on a NuPAGE^™^ 4-12% Bis-Tris Protein Gel (NP0322BOX, Thermo Fisher Scientific Inc.), and transferred to PVDF membranes (FFP24, Beyotime). The membranes were blocked with QuickBlock^™^ blocking buffer (P0222, Beyotime) in PBSTw, probed with primary antibodies, and followed by application of horseradish peroxidase-conjugated secondary antibodies [goat anti-mouse (BA1050, Wuhan Boster Biological Engineering Co., Ltd.) or goat anti-rabbit (BA1054, Wuhan Boster Biological Engineering Co., Ltd.)]. The signal was revealed by BeyoECL Plus Kit (P0018S, Wuhan Boster Biological Engineering Co., Ltd.) and imaged using an Odyssey FC imaging system (LI-COR Biosciences, Lincoln, NE, USA).

### Histopathological Analysis

For histological examination, the liver and spleen tissues were harvested and immediately fixed in 10% buffered formaldehyde (Guangzhou Chemical Reagent Factory, China) for 48 h. Five-micrometer-thick sections (50 or 100 μm distance between sections) of the organs from mice were stained with hematoxylin (1051740500, Sigma-Aldrich) and eosin (318906, Sigma-Aldrich) solution to evaluate the histological changes. Sections, blinded for groups, were histopathologically evaluated under 20× objective lens in five noncontiguous sections from 4 mice of each group. The semi-quantitative histopathological scores were determined based on previously described criteria (20, 27) with modification. In brief, the histological changes were scored as 0, 1, 2, and 3 (absent, mild inflammation, moderate inflammation and necrosis, and severe inflammation and necrosis, respectively).

### Ultrastructural Analysis

Tissue samples of livers and spleens of mice from different groups were fixed in 3% glutaraldehyde and 1% osmium tetroxide [both in 100 mM PBS, pH 7.2] before being dehydrated through a series of graded ethanol solutions. The fixed tissues were then embedded in SPI Pon 812 resin as instructed by the manufacture (02660-AB, Structure Probe Inc., West Chester, PA, USA). Ultrathin sections (70 nm) were cut from the embedded tissues using the Leica EM UC6 ultramicrotome (Leica Microsystems, Wetzlar, Germany) and mounted on formvar-coated grids. The sections were then stained for 15 min in aqueous 1% uranyl acetate followed by 0.2% lead citrate. Finally, samples were analyzed under a JEM100CX-II transmission electron microscope (JEOL Ltd., Tokyo, Japan) at an accelerating voltage of 100 kV.

### Immunohistochemical Staining

Immunohistochemistry analysis was carried out using a Strept Avidin Biotin Complex (SABC)-alkaline phosphatase (AP) kit (SA1052, Wuhan Boster Biological Engineering Co., Ltd.). Liver sections (5 μm) of mice from different groups were deparaffinized and rehydrated in distilled water, followed by inactivating endogenous peroxidases with 3% hydrogen peroxide for 10 min at 37°C. Heat-induced antigen retrieval was carried out in an 800 W microwave oven (Media, Shunde, China) for 30 min. Then, nonspecific binding was blocked by incubation in 5% bovine serum albumin in PBS (pH 7.4) for 20 min at room temperature. The sections were incubated with primary antibodies in a dilution of 1:200 overnight at 4°C, and then incubated with the goat anti-rabbit IgG secondary antibody in the kit. Sections incubated with secondary antibodies were only used as isotype controls. Signals were detected with SABC-AP reagent, and developed using BCIP/NBT reagent. Finally, the sections were counterstained with hematoxylin (1051740500, Sigma-Aldrich) and imaged under a M8 slice scanner (Precipoint, Jena, Germany). Positive cells were identified by dark-brown staining, and quantified by using Image-Pro Plus (Image Z1 software, Media Cybernetics, MD, USA). The number of cells in each field was determined under high power field as well as the area of each field (0.177 mm^2^). The density of positive cells was expressed as cell number per square millimeter.

### Immunofluorescence Assays

Liver sections (5 μm) were dewaxed firstly, and treated with antigen retrieval solution (P0088, Beyotime) according to the manufacture’s protocol. Then the sections were blocked using Immunol Staining Blocking Buffer (P0102, Beyotime) at room temperature for 60 min. The fluorescence sections were incubated with primary antibody at room temperature for 60 min in a dark chamber, incubated with fluorescence labeling secondary antibody at room temperature for 60 min in a dark chamber, and then incubated with DAPI (ab228549, Abcam) for 30 min in a dark chamber. CD137^+^ cells were marked by green fluorescent signals. The sections were observed under an EVOS FL autofluorescence microscope (Thermo Fisher Scientific Inc.), and positive cells were automatically counted by Image-pro Plus 6.0 (Media Cybernetics).

### Statistical Analysis

Statistical analysis of the data was performed using Log-rank test, Wilcoxon rank sum test, Student’s *t*-test, and one-way ANOVA followed by Bonferoni’s multiple comparison tests using IBM SPSS Statistics version 22.0 (IBM Corp., Armonk, NY, USA). Pearson’s correlation coefficient was used to analyze correlations between the levels of Gal-9, CD137, and IFNγ. All graphs were generated using GraphPad Prism 8 (GraphPad software, San Diego, CA, USA). Data are presented as mean ± standard deviation (SD) at least three independent biological replicates. A value of *P* lower than 0.05 was considered statistically significant.

## Results

### Blockage of Galectin-Receptor Interactions Increased Survival Rate and Decreased Parasite Burden of *T. gondii*-Infected Mice

**Figure 1:**
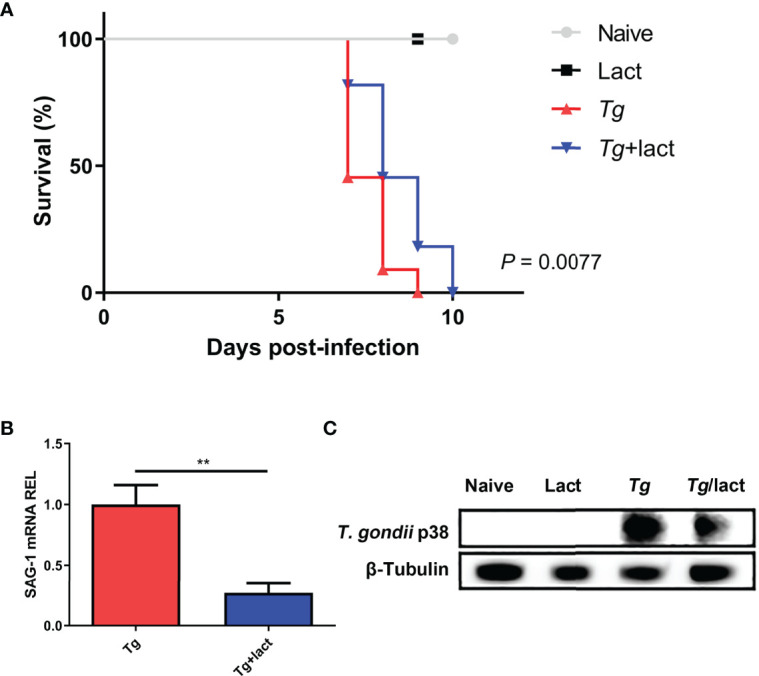
Survival rate and parasite burden. **(A)** Survival rate. *Tg-*infected mice (n = 11) died between days 7 and 9 p.i., while *Tg*+lact mice (n = 11) died between days 7 and 10 p.i. **(B)** Parasite burden in the liver tissues was estimated based on SAG1 mRNA level measured by qRT-PCR. Values are means from triplicate measurements, and data are shown as − ΔΔCt values; two independent experiments were performed with four mice per group. **P < 0.01. Y axis represents the mRNA relative expression level (REL) of different genes. **(C)** Parasite burden in the liver tissues was estimated based on the protein level of p38 from *T. gondii* detected by Western blot assay.

Mice were monitored daily after *T. gondii* infection. *Tg-*infected mice died between 7 and 9 days p.i., whereas *Tg+*lact mice died between 7 and 10 days p.i. Log-rank test showed that compared with *Tg*-infected mice, there was a significant increase of survival rate of *Tg+*lact mice (*P* < 0.01, **Figure 1A**). Compared with *Tg-*infected mice, *T. gondii* SAG1 mRNA expression level in the liver of *Tg+*lact mice was significantly decreased (*P* < 0.01) (**Figure 1B**). Western blot assay confirmed that *T. gondii* p38 protein level was decreased in the liver tissue of *Tg+*lact mice in comparison of that of *Tg-*infected mice (**Figure 1C**). The results indicated that blockage of galectin-receptor interactions may contribute to host defense against acute *T. gondii* infection.

### Blockage of Galectin-Receptor Interactions Attenuated Tissue Pathology of *T. gondii*-Infected Mice

**Figure 2:**
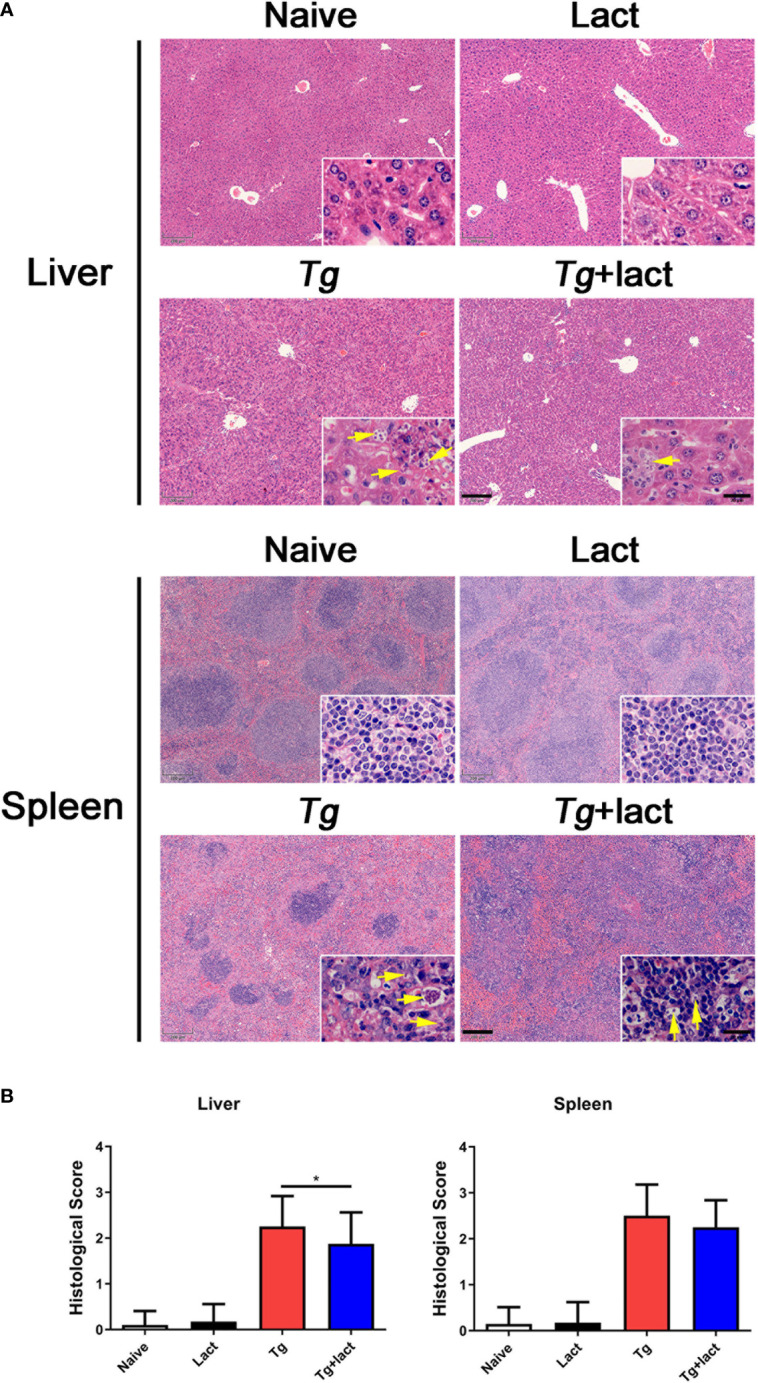
The liver and spleen histopathology of mice from different groups. Mice were i.p. inoculated with 10^2^ tachyzoites of *T. gondii* and killed at day 7 p.i. **(A)** Representative microscopic pictures showed tissue sections stained with H&E from different groups. Original magnification 50× (scale bar = 200 µm) of the large image; the small image is 1000× (scale bar = 20 μm). Yellow arrows indicate *T. gondii* tachyzoites. **(B)** Histopathological score analysis of the liver and spleen tissues. Data are represented as mean ± SD. There were 4 mice per group, and the data are representative of two experiments. *P < 0.05.

To evaluate the effects of blockage of galectin-receptor interactions on tissue pathological changes, the liver and spleen tissues of mice from different groups were examined histologically (**Figure 2A**). No histological changes were observed in the liver and spleen tissues of uninfected mice injected with PBS or α-lactose. After *T. gondii* infection, severe damage with a great number of inflammatory foci of cell infiltrates including neutrophils, lymphocytes, and mononuclear cells were observed in the liver tissue of *Tg-*infected mice. In comparison, attenuated histological damage was observed in the liver tissue of *Tg+*lact mice. Compared with *Tg-*infected mice, semi-quantitative histopathological score based on pathological changes in the liver was significantly decreased in *Tg+*lact mice (*P* < 0.05, **Figure 2B**). There were a large amount of infiltrated inflammatory cells and tachyzoites observed in the spleens of *Tg-*infected mice and *Tg+*lact mice, and lots of lymphocytes were intruded into the red pulps, which led to the boundary between the white pulp and red pulp blurred (**Figure 2A**). However, the semi-quantitative histopathological scores in the spleens between *Tg-*infected mice and *Tg+*lact mice had no significant difference (*P* > 0.05, **Figure 2B**).

**Figure 3:**
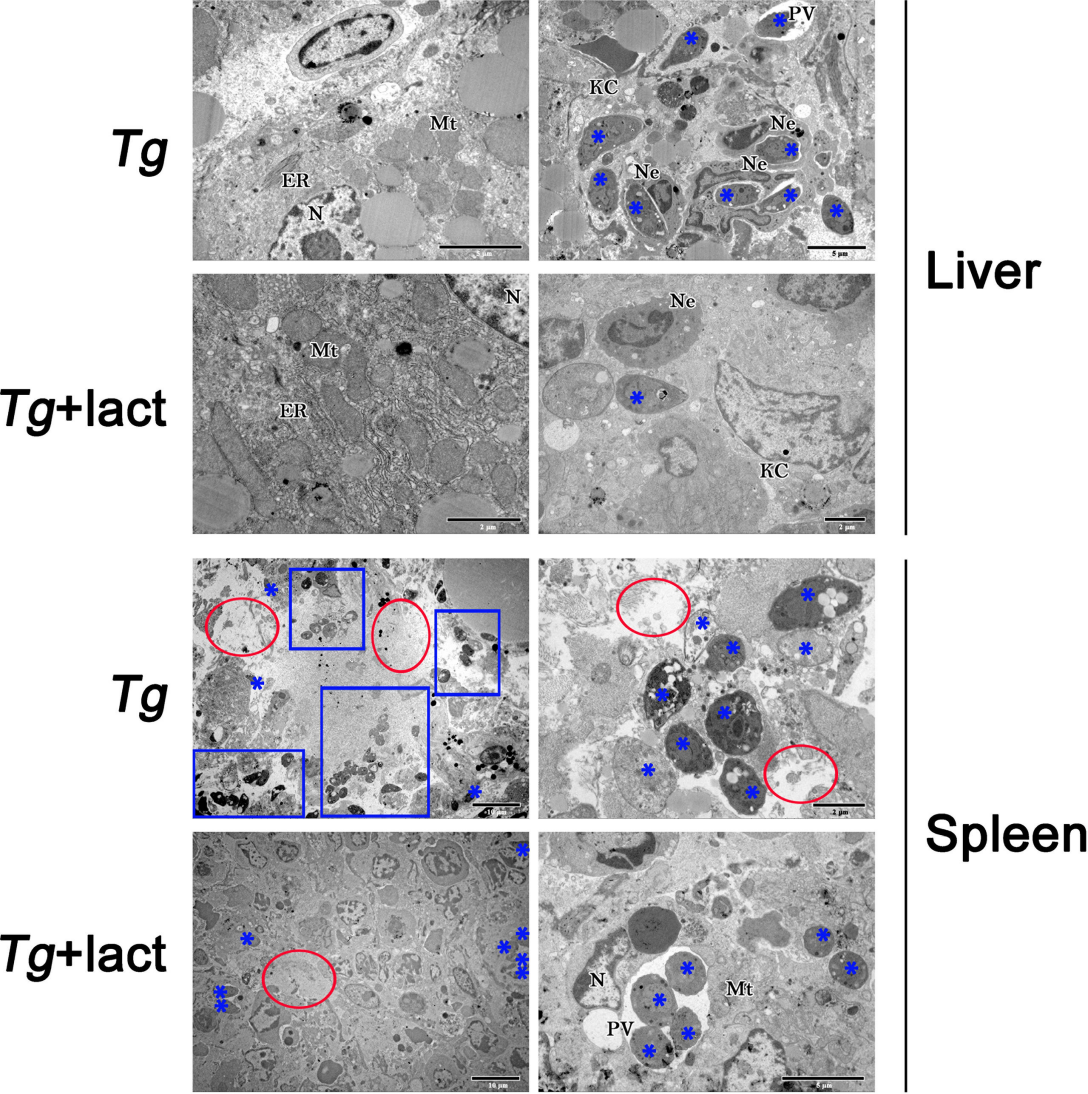
The ultrastructure of the livers and spleens in *T. gondii*-infected mice with or without α-lactose treatment. Blue stars indicate *T. gondii* tachyzoites, blue rectangles indicate the accumulation areas of tachyzoites, and red circles indicate the large necrosis areas. N, nucleus; ER, endoplasmic reticulum; Mt, mitochondrion; Ne, neutrophil; KC, Kupffer cell; PV, parasitophorous vacuole.

Transmission electron microscopy (TEM) was used to reveal the subcellular structures of the liver and spleen tissues from the two *T. gondii*-infected groups **(** **Figure 3****)**. Cellular organelles, such as mitochondria and endoplasmic reticulum (ER) in the hepatocytes of *Tg-*infected mice, became swollen and deformed. In addition, neutrophils, Kupffer cells (KCs), and numerous *T. gondii* tachyzoites were observed in the liver tissue of this group. Similar subcellular structure changes of hepatocytes were observed in the liver of *Tg+*lact mice, however, the lesion was less severe and fewer *T. gondii* tachyzoites were observed in the liver tissue. In the necrosis area of spleen tissue of *Tg-*infected mice, cell membranes and intercellular connections were damaged, and normal host cells were hardly observed. In addition, there were numerous extracellular *T. gondii* tachyzoites observed by TEM. However, the damage of ultrastructure of the spleen tissue of *Tg+*lact mice was less severe. The number of tachyzoites was fewer, and most of them were inside intracellular parasitophorous vacuoles (PVs) of the spleen tissue cells rather than egressed into extracellular space of the tissue.

### Blockage of Galectin-Receptor Interactions Increased CD137 Expression in the Liver of *T. gondii*-Infected Mice

**Figure 4:**
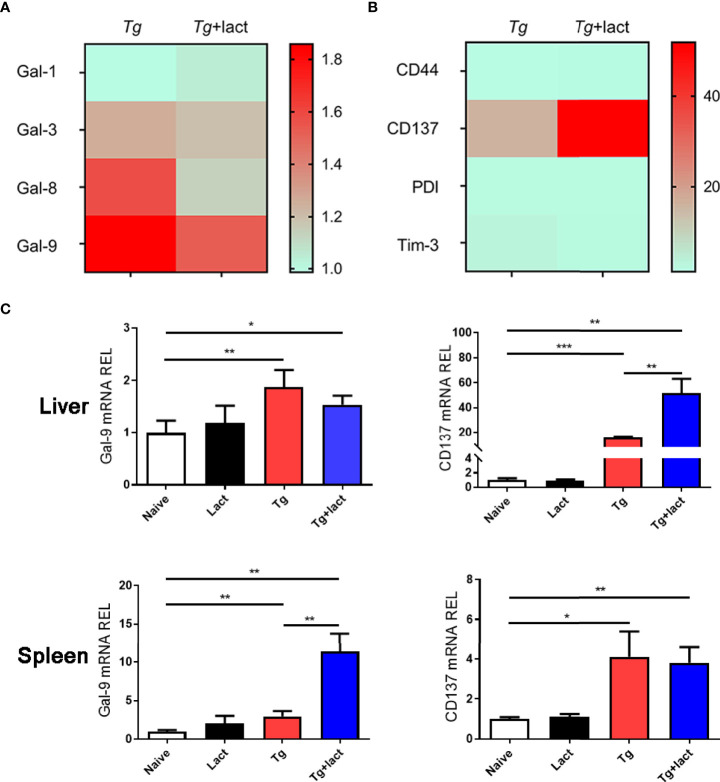
Gal-9 and its receptor CD137 were upregulated during *T. gondii* infection. Heatmap visualization showed the mRNA expressions of Gal-1, Gal-3, Gal-8, and Gal-9 **(A)** and the receptors of Gal-9 (CD44, CD137, PDI, and Tim-3) **(B)** in the livers of *Tg*-infected mice and *Tg*+lact mice, while histograms showed the expression levels of Gal-9 and CD137 in the livers and spleens of mice from different groups **(C)**. Liver and spleen tissues were collected on day 7 p.i. for evaluating mRNA expression levels by using qRT-PCR. Values are means from triplicate measurements, and data are presented as means ± SD; two independent experiments were performed with four mice per group. *P < 0.05, **P < 0.01, and ***P < 0.001. Y axis represents the mRNA relative expression level (REL) of different genes.

The mRNA expression levels of Gal-1, Gal-3, Gal-8, and Gal-9 (**Figure 4A**), and Gal-9 receptors (CD44, CD137, PDI, and Tim-3) (**Figure 4B**) in the livers of *Tg*-infected mice and *Tg*+lact mice were examined by using qRT-PCR and presented as heatmaps. The most conspicuous upregulation values were found for Gal-9 and its receptor CD137. Compared with uninfected mice, the mRNA levels of Gal-9 (*P* < 0.01) and CD137 (*P* < 0.001 and *P* < 0.05, respectively) were significantly increased in the liver and spleen of *Tg-*infected mice, and the levels of Gal-9 (*P* < 0.05 and *P* < 0.01, respectively) and CD137 (*P* < 0.01) were significantly increased in the liver and spleen of *Tg*+lact mice. Compared with *Tg-*infected mice, the mRNA levels of CD137 in the liver and Gal-9 in the spleen of *Tg*+lact mice were significantly increased (*P* < 0.01, **Figure 4C**).

**Figure 5:**
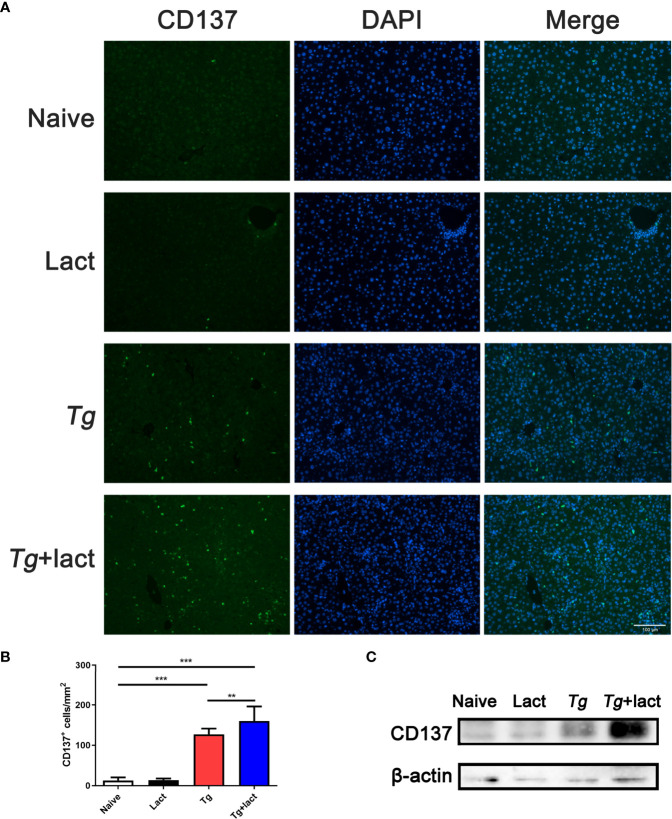
CD137^+^ cells in the liver tissues of mice from different groups. **(A)** Immunofluorescence staining showed CD137^+^ cells (labeled green) in the livers of mice. Original magnification 200× (scale bar = 100 µm). **(B)** Morphometric analysis in liver tissues by showing the number of CD137^+^ cells per square millimeter. Data are presented as means ± SD; experiments were performed with four mice per group. **P < 0.01, and ***P < 0.001. **(C)** The protein levels of CD137 in the livers of mice were detected by Western blot assay.

Immunofluorescence assay showed that there were only a few of CD137^+^ cells observed in the liver tissue of uninfected mice (**Figure 5A**). However, compared with uninfected mice, the numbers of CD137^+^ cells in the livers of both *Tg*-infected mice and *Tg+*lact mice were significantly increased (*P* < 0.001). Compared with *Tg*-infected mice, the number of CD137^+^ cells in the liver of *Tg+*lact mice was significantly increased (*P* < 0.01, **Figure 5B**). Western blot assay confirmed that the protein level of CD137 in the liver tissue of *Tg+*lact mice was increased compared with that of *Tg-*infected mice (**Figure 5C**).

### Blockage of Galectin-Receptor Interactions Up-Regulated mRNA Expressions of IFNγ, IL-4, and IL-10 in the Liver of *T. gondii*-Infected Mice

**Figure 6:**
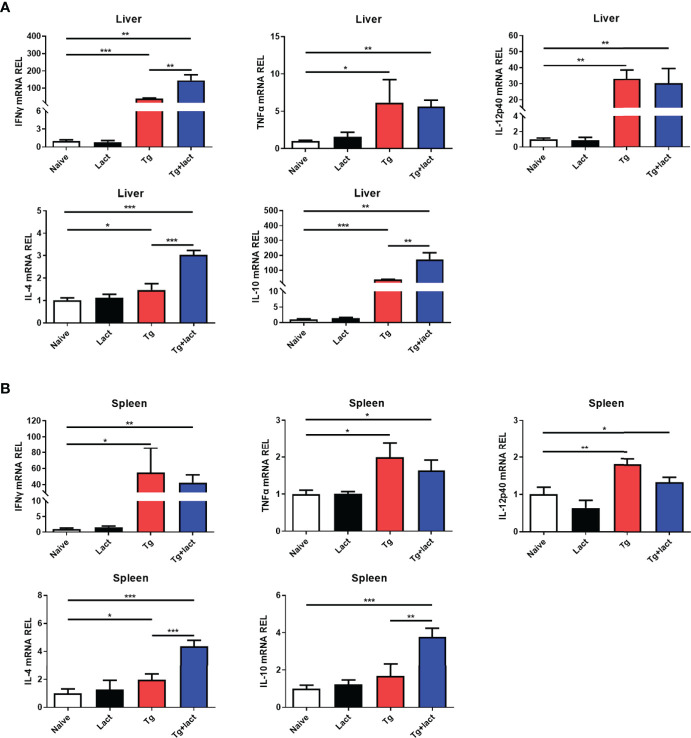
The mRNA expression levels of Th1 and Th2 cytokines in the livers **(A)** and spleens **(B)** of mice from different groups on day 7 p.i. Values are means from triplicate measurements, and data are presented as means ± SD; two independent experiments were performed with four mice per group. *P < 0.05, **P < 0.01, and ***P < 0.001. Y axis represents the mRNA relative expression level (REL) of different genes.

The effects of α-lactose treatment on Th1 and Th2 cytokine responses during *T. gondii* infection were evaluated by measuring the mRNA expression levels of IFNγ, TNFα, IL-12p40, IL-4, and IL-10 in the livers and spleens of mice from different groups. Compared with uninfected mice, there were significantly increased mRNA expression levels of IFNγ (*P* < 0.001 and *P* < 0.05, respectively), TNFα (*P* < 0.05), IL-12p40 (*P* < 0.01), and IL-4 (*P* < 0.05) in the liver and spleen of *Tg*-infected mice, and significantly increased IL-10 level (*P* < 0.001) in the liver of *Tg*-infected mice on day 7 p.i.; significantly increased levels of IFNγ (*P* < 0.01), TNFα (*P* < 0.01 and *P* < 0.05, respectively), IL-12p40 (*P* < 0.01 and *P* < 0.05, respectively), IL-4 (*P* < 0.001), and IL-10 (*P* < 0.01 and *P* < 0.001, respectively) in the liver and spleen of *Tg*+lact mice on day 7 p.i. However, compared with *Tg*-infected mice, there were significantly increased IFNγ level (*P* < 0.01) in the liver, and significantly increased levels of IL-4 (*P* < 0.001) and IL-10 (*P* < 0.01) in the liver and spleen of *Tg*+lact mice on day 7 p.i. The results indicated that blockage of galectin-receptor interactions may increase both Th1 and Th2 responses in the liver of *T. gondii*-infected mice (**Figure 6**).

### Correlations Between Gal-9 and CD137, Gal-9 and IFNγ, and CD137 and IFNγ in the Liver and Spleen of *Tg*-Infected Mice or *Tg*+Lact Mice

**Figure 7:**
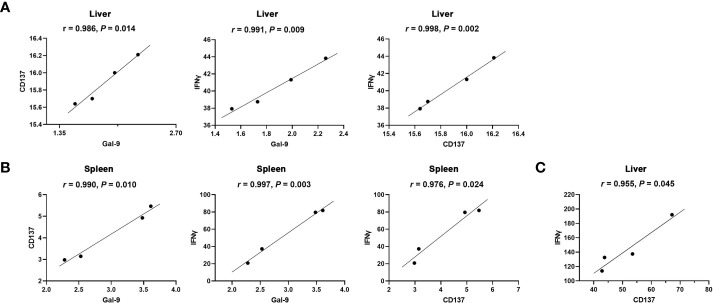
Correlation analysis between the mRNA expression levels detected in the livers and spleens of *Tg*-infected mice or *Tg*+lact mice (n = 4). **(A)** Significant correlations between Gal-9 and CD137, Gal-9 and IFNγ, and CD137 and IFNγ in the liver, and **(B)** Significant correlations between Gal-9 and CD137, Gal-9 and IFNγ, and CD137 and IFNγ in the spleen of *Tg*-infected mice. **(C)** Significant correlation between CD137 and IFNγ in the liver of *Tg*+lact mice. The *r* value generates for the theoretical line of best fit, while the *P* value indicates the significance of the correlation.

The correlations between the mRNA levels of Gal-9 and CD137, Gal-9 and IFNγ, and CD137 and IFNγ in the liver and spleen of *Tg*-infected mice or *Tg*+lact mice were evaluated, herein only significant correlations were presented. In *Tg*-infected mice, there were significant correlations between the mRNA levels of Gal-9 and CD137 (*r* = 0.986, *P* = 0.014), Gal-9 and IFNγ (*r* = 0.991, *P* = 0.009), and CD137 and IFNγ (*r* = 0.998, *P* = 0.002) in the liver (**Figure 7A**), and significant positive correlations between the mRNA levels of Gal-9 and CD137 (*r* = 0.990, *P* = 0.010), Gal-9 and IFNγ (*r* = 0.997, *P* = 0.003), and CD137 and IFNγ (*r* = 0.976, *P* = 0.024) in the spleen (**Figure 7B****)**. In *Tg*+lact mice, there was significant correlation between the mRNA levels of CD137 and IFNγ (*r* = 0.955, *P* = 0.045) in the liver (**Figure 7C**).

### Blockage of Galectin-Receptor Interactions Promoted M2 Macrophage Polarization in the Liver of *T. gondii*-Infected Mice

**Figure 8:**
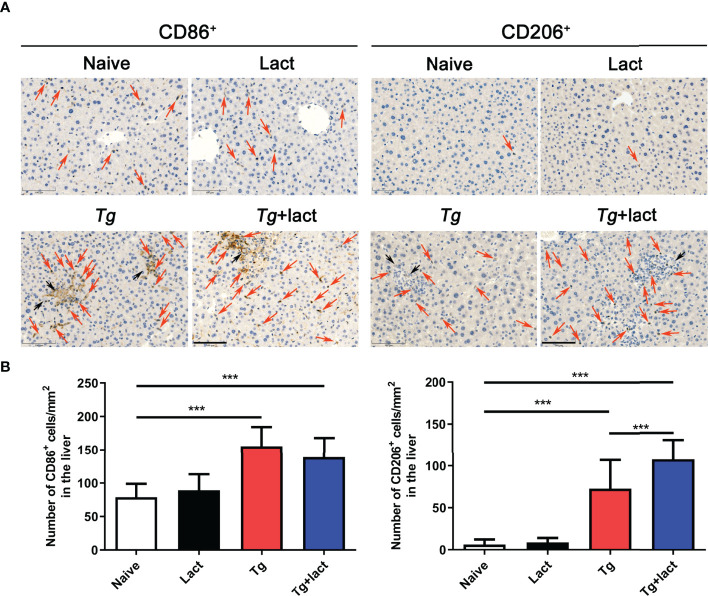
Immunohistochemical staining for CD86^+^ and CD206^+^ cells in the livers of mice from different groups. **(A)** Immunohistochemical staining of CD86^+^ and CD206^+^ cells were labeled by CD86 mAb and CD206 pAb, respectively. Red arrows indicate positive cells and black arrows indicate tachyzoites. Original magnification 200× (scale bar = 100 µm). **(B)** Morphometric analysis showed the number of CD86^+^ or CD206^+^ cells per square millimeter in the liver tissues. Data are presented as means ± SD; experiments were performed with four mice per group. ***P < 0.001.

Macrophages are a major player in defense against pathogens in the liver. In uninfected mice, only a few macrophages (stained brown) were observed in the liver tissue when using CD86 and CD206 as the immunohistochemical labels for M1 and M2 macrophages, respectively (**Figure 8A**). Compared with uninfected mice, the numbers of CD86^+^ macrophages in the livers of *Tg*-infected and *Tg*+lact mice were significantly increased (*P* < 0.001). However, there was no significant difference between the two *T. gondii*-infected groups (*P* > 0.05). Compared with uninfected mice, the numbers of CD206^+^ macrophages in the livers of *Tg*-infected mice and *Tg*+lact mice were significantly increased (*P* < 0.001). Compared with *Tg-*infected mice, the number of CD206^+^ macrophages in the liver of *Tg+*lact mice was significantly increased (*P <* 0.001, **Figure 8B**). The results indicated that blockage of galectin-receptor interactions may induce M2 macrophage polarization in the liver of *T. gondii*-infected mice.

## Discussion

As an opportunistic protozoan, chronic infection with *T. gondii* is likely one of the most common infections in humans. Most *T. gondii* infections show minimal symptoms, but immunocompromised patients tend to have a poor prognosis (28). Clinical disease can also occur in immunocompetent adults, in particular manifesting as ocular toxoplasmosis (29). In liver, *T. gondii* has been associated with a number of pathological changes including hepatomegaly, granuloma, hepatitis, and necrosis (5, 21, 30, 31). A case report showed that a child suffered from acquired toxoplasmosis with a rare manifestation of severe liver damage (32). In the present study, our data showed that blockage of galectin-receptor interactions could prolong the survival time, decrease liver parasite burden, and attenuate liver pathology of *T. gondii*-infected mice.

*T. gondii* infection induces Th1-biased immune response, which is crucial for resistance against toxoplasmosis (33). It has been reported that extensive liver damage is accompanied the elevated levels of Th1 cytokines (34). In the present study, *T. gondii*-infected C57BL/6 mice showed high parasite burden and strong inflammatory outcome in the liver, accompanied with a dominant Th1 response characterized with high expression levels of Th1 cytokines (IFNγ, TNFα, and IL-12p40) in the liver tissue after *T. gondii* infection; while *T. gondii-*infected mice treated with α-lactose showed prolonged survival time, decreased tissue parasite burden, and attenuated liver histopathology, accompanied with higher mRNA expression levels of CD137, IFNγ, IL-4, and IL-10, and enhanced M2 macrophage polarization in the liver. It has been reported that increased IFNγ production may effectively promote parasite clearance for its pivotal role in driving immune responses against *T. gondii* (35, 36). IL-4 and IL-10 can decrease parasite load in *T. gondii*-infected mice treated with the mast cell degranulation inhibitor disodium cromoglycate (8), and IL-10 is required for control of necrosis and immune-mediated pathology during *T. gondii* infection (37–39). M1 and M2 macrophages can be converted into each other in certain microenvironments (40), and M2 phenotype macrophages are believed to play important roles in control of parasites, such as *Trypanosoma cruzi, Trichinella spiralis*, and *Schistosoma mansoni*, by producing IL-4, IL-10, chitinase-3-like protein 3 (Ym1), and arginase-1 (Arg-1) (41–43). Previous study by our group has demonstrated the important role of M2 phenotype markers Arg-1 and Ym1 during toxoplasmic encephalitis (TE) in *T. gondii*-susceptible C57BL/6 mice (44). *Toxo*ROP16_I/III_ promotes the polarization of M2 cells, and enhances the synthesis of Arg-1, IL-10, transforming growth factor-β1, and IL-13, which may ameliorate the pathogenesis of inflammatory bowel disease in an *in vitro* experimental model (45). In this study, the increased IFNγ, IL-4, and IL-10 expression levels, and increased M2 macrophage numbers induced by blockage of galectin-receptor interactions may be one of the reasons that *Tg*+lact mice had lower parasite burden and attenuated liver histopathology than those of *Tg*-infected mice. Our data suggested that blockage of galectin-receptor interactions may have a potential beneficial effect on acute *T. gondii* infection.

Galectins, a conserved group of immunomodulatory animal lectins, are widely expressed in different tissues and a number of immune cell populations (46), and are involved in lots of physiological functions, such as inflammation, immune responses, cell migration, autophagy, and signaling (47). So far 15 galectins have been identified in mammals (48). Gal-9 is widely expressed in various tissues such as spleen, lung, heart, and thymus, and is particularly abundant in the liver tissue (49). Gal-9 involves in a variety of pathological processes including autoimmunity, fibrosis, and cancer (50), as well as regulation of inflammation (24, 49–51). Studies in our group have found that galectins may contribute to severe egg granulomatous inflammation and tissue fibrosis in the liver, spleen, and large intestine of *Schistosoma japonicum*-infected mice (52). Both Gal-3 and Gal-9 are important factors in TE-susceptible C57BL/6 mice and TE-resistant BALB/c mice infected with *T. gondii* Prugniaud strain (44). Blockage of galectin-receptor interactions could lead to elevated heart eosinophil recruitment, exacerbated heart pathology and fibrosis, and functional damage to the heart during acute *T. spiralis* infection in a murine model (53). In addition, blockage of galectin-receptor interactions leads to decreased host survival rate, increased peripheral blood parasitemia, increased parasite burden in the liver, and increased CD68^+^ macrophages and exacerbated liver pathology of *Plasmodium berghei*-infected mice (54). However, Gal-9 was also found to be upregulated in inflamed tissues, suggesting that Gal-9 may be closely related to inflammatory development under infectious or autoimmune conditions (19, 55). In the present study, the mRNA expression levels of Gal-1, Gal-3, Gal-8, and Gal-9 were measured in the liver of *T. gondii*-infected mice, and Gal-9 showed the most conspicuous upregulation. Our data revealed that blocking galectin-receptor interactions exhibited decreased parasite burden, elevated M2 macrophage population, up-regulated Th2 cytokines, and alleviated liver pathology in *T. gondii*-infected mice. Therefore, Gal-9 may play a diverse role during acute *T. gondii* infection, and blockage of Gal-9-receptor interaction may have a potential role for attenuating toxoplasmic liver damage.

Among the receptors of Gal-9, Tim-3 has been well studied for its critical role in the immune regulation during *T. gondii* infection (27, 56, 57). However, the other three receptors, CD137 (14), CD44 (15), and PDI (16) are rarely investigated. In the present study, our data showed that the upregulation value of CD137 was markedly higher than those of CD44, PDI, and Tim-3 in the liver of *T. gondii*-infected mice. In addition, the mRNA level and protein level of CD137, and the numbers of CD137 positive cells in the liver of *Tg*+lact mice were all higher than those of *Tg*-infected mice. As an important co-stimulatory molecule functioning in T cells, dendritic cells, and natural killer cells (14, 58), CD137 preferentially induces Th1 response by increasing IFNγ and CD8^+^ T cell proliferation through interacting with CD137L (59, 60). Our previous data showed upregulation of CD137 expression in experimental ocular toxoplasmosis (20). Here, our results further demonstrated the positive correlation between CD137 and IFNγ in the livers of *Tg*-infected mice and *Tg*+lact mice, suggesting that Gal-9-CD137 pathway may regulate T cell immune responses during acute *T. gondii* infection.

In conclusion, our study has provided evidence that galectin-receptor interactions, especially Gal-9-CD137 signaling, is important for the regulation of T cell and macrophage functions, which may play a role during *T. gondii* infection. Further studies are necessary for a more detailed understanding of the mechanisms.

## Data Availability Statement

The original contributions presented in the study are included in the article/supplementary material. Further inquiries can be directed to the corresponding author.

## Ethics Statement

Animal studies were conducted according to protocols approved by the Animal Experimentation Ethics Committee of Zhongshan School of Medicine on Laboratory Animal Care at Sun Yat-sen University, China.

## Author Contributions

FL conceived and designed the experiments. JH and YH performed the experiments. JH and FL analyzed the data. FL and JH wrote the manuscript. All authors approved the submitted version.

## Funding

This work was supported by the National Natural Science Foundation of China (81971955), the Natural Science Foundation of Guangdong Province (2019A1515011667, 2021A1515012115), and 2021 Graduate Education Innovation Plan Project of Guangdong Province (2021SFKC003).

## Conflict of Interest

The authors declare that the research was conducted in the absence of any commercial or financial relationships that could be construed as a potential conflict of interest.

## Publisher’s Note

All claims expressed in this article are solely those of the authors and do not necessarily represent those of their affiliated organizations, or those of the publisher, the editors and the reviewers. Any product that may be evaluated in this article, or claim that may be made by its manufacturer, is not guaranteed or endorsed by the publisher.

## References

[1] Reid AJ Vermont SJ Cotton JA Harris D Hill-Cawthorne GA Konen-Waisman S. Comparative Genomics of the Apicomplexan Parasites *Toxoplasma Gondii* and *Neospora Caninum*: Coccidia Differing in Host Range and Transmission Strategy. PLoS Pathog (2012) 8(3):22. doi: 10.1371/journal.ppat.1002567 PMC3310773 22457617

[2] Milne G Webster JP Walker M. *Toxoplasma Gondii*: An Underestimated Threat? Trends Parasitol (2020) 36(12):959–69. doi: 10.1016/j.pt.2020.08.005 33012669

[3] Waldman BS Schwarz D Wadsworth MH 2nd Saeij JP Shalek AK Lourido S. Identification of a Master Regulator of Differentiation in *Toxoplasma* . Cell (2020) 180(2):359–72.e316. doi: 10.1016/j.cell.2019.12.013 31955846 PMC6978799

[4] Marra CM. Central Nervous System Infection With *Toxoplasma Gondii* . Handb Clin Neurol (2018) 152:117–22. doi: 10.1016/B978-0-444-63849-6.00009-8 29604970

[5] Doğan N Kabukçuoğlu S Vardareli E. Toxoplasmic Hepatitis in an Immunocompetent Patient. Turkiye Parazitol Derg (2007) 31(4):260–3. 18224612

[6] Pazoki H Ziaee M Anvari D Rezaei F Ahmadpour E Haghparast-kenari B. *Toxoplasma Gondii* Infection as a Potential Risk for Chronic Liver Diseases: A Systematic Review and Meta-Analysis. Microb Pathog (2020) 149:104578. doi: 10.1016/j.micpath.2020.104578 33069795

[7] Dupont CD Christian DA Hunter CA. Immune Response and Immunopathology During Toxoplasmosis. Semin Immunopathol (2012) 34(6):793–813. doi: 10.1007/s00281-012-0339-3 22955326 PMC3498595

[8] Huang B Huang S Chen Y Zheng H Shen J Lun ZR. Mast Cells Modulate Acute Toxoplasmosis in Murine Models. PLoS One (2013) 8(10):e77327. doi: 10.1371/journal.pone.0077327 24146978 PMC3797692

[9] Barondes SH Cooper DN Gitt MA Leffler H. Galectins. Structure and Function of a Large Family of Animal Lectins. J Biol Chem (1994) 269(33):20807–10. doi: 10.1016/s0021-9258(17)31891-4 8063692

[10] Liu FT Rabinovich GA. Galectins: Regulators of Acute and Chronic Inflammation. Ann N Y Acad Sci (2010) 1183:158–82. doi: 10.1111/j.1749-6632.2009.05131.x 20146714

[11] Moar P Tandon R. Galectin-9 as a Biomarker of Disease Severity. Cell Immunol (2021) 361:104287. doi: 10.1016/j.cellimm.2021.104287 33494007

[12] Sabatos CA Chakravarti S Cha E Schubart A Sanchez-Fueyo A Zheng XX. Interaction of Tim-3 and Tim-3 Ligand Regulates T Helper Type 1 Responses and Induction of Peripheral Tolerance. Nat Immunol (2003) 4(11):1102–10. doi: 10.1038/ni988 14556006

[13] Zhu C Anderson AC Schubart A Xiong H Imitola J Khoury SJ. The Tim-3 Ligand Galectin-9 Negatively Regulates T Helper Type 1 Immunity. Nat Immunol (2005) 6(12):1245–52. doi: 10.1038/ni1271 16286920

[14] Madireddi S Eun SY Lee SW Nemcovicova I Mehta AK Zajonc DM. Galectin-9 Controls the Therapeutic Activity of 4-1BB-Targeting Antibodies. J Exp Med (2014) 211(7):1433–48. doi: 10.1084/jem.20132687 PMC4076583 24958847

[15] Wu C Thalhamer T Franca RF Xiao S Wang C Hotta C. Galectin-9-CD44 Interaction Enhances Stability and Function of Adaptive Regulatory T Cells. Immunity (2014) 41(2):270–82. doi: 10.1016/j.immuni.2014.06.011 PMC4219323 25065622

[16] Bi S Hong PW Lee B Baum LG. Galectin-9 Binding to Cell Surface Protein Disulfide Isomerase Regulates the Redox Environment to Enhance T-Cell Migration and HIV Entry. Proc Natl Acad Sci USA (2011) 108(26):10650–5. doi: 10.1073/pnas.1017954108 PMC3127870 21670307

[17] Thijssen VL Hulsmans S Griffioen AW. The Galectin Profile of the Endothelium: Altered Expression and Localization in Activated and Tumor Endothelial Cells. Am J Pathol (2008) 172(2):545–53. doi: 10.2353/ajpath.2008.070938 PMC2312370 18202194

[18] Katoh S Ishii N Nobumoto A Takeshita K Dai SY Shinonaga R. Galectin-9 Inhibits CD44-Hyaluronan Interaction and Suppresses a Murine Model of Allergic Asthma. Am J Respir Crit Care Med (2007) 176(1):27–35. doi: 10.1164/rccm.200608-1243OC 17446336

[19] Schaefer K Webb NE Pang M Hernandez-Davies JE Lee KP Gonzalez P. Galectin-9 Binds to O-Glycans on Protein Disulfide Isomerase. Glycobiology (2017) 27(9):878–87. doi: 10.1093/glycob/cwx065 PMC5881768 28810662

[20] Chen SJ Zhang YX Huang SG Lu FL. Galectins Expressed Differently in Genetically Susceptible C57BL/6 and Resistant BALB/c Mice During Acute Ocular *Toxoplasma Gondii* Infection. Parasitology (2017) 144(8):1064–72. doi: 10.1017/S0031182017000270 28274286

[21] Ortego TJ Robey B Morrison D Chan C. Toxoplasmic Chorioretinitis and Hepatic Granulomas. Am J Gastroenterol (1990) 85(10):1418–20. 2220741

[22] Singh S Lodha R Passi GR Bhan MK. Cholestatic Jaundice Due to Congenital *Toxoplasma Gondii* Infection. Indian J Pediatr (1998) 65(1):154–7. doi: 10.1007/BF02849711 10771960

[23] Ustun S Aksoy U Dagci H Ersoz G. Incidence of Toxoplasmosis in Patients With Cirrhosis. World J Gastroenterol (2004) 10(3):452–4. doi: 10.3748/wjg.v10.i3.452 PMC4724918 14760779

[24] Golden-Mason L Rosen HR. Galectin-9: Diverse Roles in Hepatic Immune Homeostasis and Inflammation. Hepatology (2017) 66(1):271–9. doi: 10.1002/hep.29106 PMC5521806 28195343

[25] Sehrawat S Reddy PBJ Rajasagi N Suryawanshi A Hirashima M Rouse BT. Galectin-9/TIM-3 Interaction Regulates Virus-Specific Primary and Memory CD8 T Cell Response. PLoS Pathog (2010) 6(5):e1000882. doi: 10.1371/journal.ppat.1000882 20463811 PMC2865527

[26] Vega-Carrascal I Reeves EP McElvaney NG. The Role of TIM-Containing Molecules in Airway Disease and Their Potential as Therapeutic Targets. J Inflamm Res (2012) 5:77–87. doi: 10.2147/jir.s34225 22952413 PMC3430008

[27] Wu B Huang B Chen Y Li S Yan J Zheng H. Upregulated Expression of Tim-3 Involved in the Process of Toxoplasmic Encephalitis in Mouse Model. Parasitol Res (2013) 112(7):2511–21. doi: 10.1007/s00436-013-3416-1 23595213

[28] Zhou Z Ortiz Lopez HIA Pérez GE Burgos LM Farina JM Saldarriaga C. Toxoplasmosis and the Heart. Curr Probl Cardiol (2021) 46(3):100741. doi: 10.1016/j.cpcardiol.2020.100741 33183832

[29] Weiss LM Dubey JP. Toxoplasmosis: A History of Clinical Observations. Int J Parasitol (2009) 39(8):895–901. doi: 10.1016/j.ijpara.2009.02.004 19217908 PMC2704023

[30] Hassan MM Farghaly AM Gaber NS Nageeb HF Hegab MH Galal N. Parasitic Causes of Hepatomegaly in Children. J Egypt Soc Parasitol (1996) 26(1):177–89. 8721239

[31] Mastroianni A Coronado O Scarani P Manfredi R Chiodo F. Liver Toxoplasmosis and Acquired Immunodeficiency Syndrome. Recenti Prog Med (1996) 87(7-8):353–5. 8831254

[32] Garty I Tal I Kaynan A. Tc-99m Colloid Lung Uptake in a Rare Case of Toxoplasmosis With Liver Involvement. Clin Nucl Med (1984) 9(6):310–3. doi: 10.1097/00003072-198406000-00002 6590161

[33] Suzuki Y Remington JS. The Effect of Anti-IFN-Gamma Antibody on the Protective Effect of Lyt-2+ Immune T Cells Against Toxoplasmosis in Mice. J Immunol (1990) 144(5):1954–6. 2106557

[34] Mordue DG Monroy F La Regina M Dinarello CA Sibley LD. Acute Toxoplasmosis Leads to Lethal Overproduction of Th1 Cytokines. J Immunol (2001) 167(8):4574–84. doi: 10.4049/jimmunol.167.8.4574 11591786

[35] Niedelman W Sprokholt JK Clough B Frickel EM Saeij JP. Cell Death of Gamma Interferon-Stimulated Human Fibroblasts Upon *Toxoplasma Gondii* Infection Induces Early Parasite Egress and Limits Parasite Replication. Infect Immun (2013) 81(12):4341–9. doi: 10.1128/IAI.00416-13 PMC3837980 24042117

[36] Rommereim LM Fox BA Butler KL Cantillana V Taylor GA Bzik DJ. Rhoptry and Dense Granule Secreted Effectors Regulate CD8(^+^) T Cell Recognition of *Toxoplasma Gondii* Infected Host Cells. Front Immunol (2019) 10:2104. doi: 10.3389/fimmu.2019.02104 31555296 PMC6742963

[37] Lu F Huang S Kasper LH. Interleukin-10 and Pathogenesis of Murine Ocular Toxoplasmosis. Infect Immun (2003) 71(12):7159–63. doi: 10.1128/IAI.71.12.7159-7163.2003 PMC308941 14638808

[38] Suzuki Y Sher A Yap G Park D Neyer LE Liesenfeld O. IL-10 is Required for Prevention of Necrosis in the Small Intestine and Mortality in Both Genetically Resistant BALB/c and Susceptible C57BL/6 Mice Following Peroral Infection With *Toxoplasma Gondii* . J Immunol (2000) 164(10):5375–82. doi: 10.4049/jimmunol.164.10.5375 10799901

[39] Wille U Nishi M Lieberman L Wilson EH Roos DS Hunter CA. IL-10 is Not Required to Prevent Immune Hyperactivity During Memory Responses to *Toxoplasma Gondii* . Parasit Immunol (2004) 26(5):229–36. doi: 10.1111/j.0141-9838.2004.00704.x 15491472

[40] Galván-Peña S O'Neill LA. Metabolic Reprograming in Macrophage Polarization. Front Immunol (2014) 5:420. doi: 10.3389/fimmu.2014.00420 25228902 PMC4151090

[41] Dzik JM Gołos B Jagielska E Zielinski Z Wałajtys-Rode E. A Non-Classical Type of Alveolar Macrophage Response to *Trichinella Spiralis* Infection. Parasit Immunol (2004) 26(4):197–205. doi: 10.1111/j.0141-9838.2004.00700.x 15367297

[42] Herbert DR Hölscher C Mohrs M Arendse B Schwegmann A Radwanska M. Alternative Macrophage Activation is Essential for Survival During Schistosomiasis and Downmodulates T Helper 1 Responses and Immunopathology. Immunity (2004) 20(5):623–35. doi: 10.1016/S1074-7613(04)00107-4 15142530

[43] Roffê E Rothfuchs AG Santiago HC Marino AP Ribeiro-Gomes FL Eckhaus M. IL-10 Limits Parasite Burden and Protects Against Fatal Myocarditis in a Mouse Model of *Trypanosoma Cruzi* Infection. J Immunol (2012) 188(2):649–60. doi: 10.4049/jimmunol.1003845 PMC3253255 22156594

[44] Liu J Huang S Lu F. Galectin-3 and Galectin-9 May Differently Regulate the Expressions of Microglial M1/M2 Markers and T Helper 1/Th2 Cytokines in the Brains of Genetically Susceptible C57BL/6 and Resistant BALB/c Mice Following Peroral Infection With *Toxoplasma Gondii* . Front Immunol (2018) 9:1648. doi: 10.3389/fimmu.2018.01648 30108583 PMC6080610

[45] Xu YW Xing RX Zhang WH Li L Wu Y Hu J. *Toxoplasma* ROP16(I/III) Ameliorated Inflammatory Bowel Diseases *via* Inducing M2 Phenotype of Macrophages. World J Gastroenterol (2019) 25(45):6634–52. doi: 10.3748/wjg.v25.i45.6634 PMC6906210 31832003

[46] Vasta GR. Galectins as Pattern Recognition Receptors: Structure, Function, and Evolution. Adv Exp Med Biol (2012) 946:21–36. doi: 10.1007/978-1-4614-0106-3_2 21948360 PMC3429938

[47] Johannes L Jacob R Leffler H. Galectins at a Glance. J Cell Sci (2018) 131(9):jcs208884. doi: 10.1242/jcs.208884 29717004

[48] van Kooyk Y Rabinovich GA. Protein-Glycan Interactions in the Control of Innate and Adaptive Immune Responses. Nat Immunol (2008) 9(6):593–601. doi: 10.1038/ni.f.203 18490910

[49] Bacigalupo ML Manzi M Rabinovich GA Troncoso MF. Hierarchical and Selective Roles of Galectins in Hepatocarcinogenesis, Liver Fibrosis and Inflammation of Hepatocellular Carcinoma. World J Gastroenterol (2013) 19(47):8831–49. doi: 10.3748/wjg.v19.i47.8831 PMC3870534 24379606

[50] Rabinovich GA Croci DO. Regulatory Circuits Mediated by Lectin-Glycan Interactions in Autoimmunity and Cancer. Immunity (2012) 36(3):322–35. doi: 10.1016/j.immuni.2012.03.004 22444630

[51] Bellac CL Coimbra RS Simon F Imboden H Leib SL. Gene and Protein Expression of Galectin-3 and Galectin-9 in Experimental Pneumococcal Meningitis. Neurobiol Dis (2007) 28(2):175–83. doi: 10.1016/j.nbd.2007.07.005 17706429

[52] Ye Z Huang S Zhang Y Mei X Zheng H Li M. Galectins, Eosinophiles, and Macrophages May Contribute to *Schistosoma Japonicum* Egg-Induced Immunopathology in a Mouse Model. Front Immunol (2020) 11:146. doi: 10.3389/fimmu.2020.00146 32231658 PMC7082360

[53] Yan J Huang S Lu F. Galectin-Receptor Interactions Regulates Cardiac Pathology Caused by *Trichinella Spiralis* Infection. Front Immunol (2021) 12:639260. doi: 10.3389/fimmu.2021.639260 34093526 PMC8175896

[54] Wu Y Huang S Xiao S He J Lu F. Impact of Galectin-Receptor Interactions on Liver Pathology During the Erythrocytic Stage of *Plasmodium Berghei* Malaria. Front Immunol (2021) 12:758052. doi: 10.3389/fimmu.2021.758052 34899708 PMC8652201

[55] Chen HY Wu YF Chou FC Wu YH Yeh LT Lin KI. Intracellular Galectin-9 Enhances Proximal TCR Signaling and Potentiates Autoimmune Diseases. J Immunol (2020) 204(5):1158–72. doi: 10.4049/jimmunol.1901114 31969388

[56] Berrocal Almanza LC Muñoz M Kühl AA Kamradt T Heimesaat MM Liesenfeld O. Tim-3 Is Differently Expressed in Genetically Susceptible C57BL/6 and Resistant BALB/c Mice During Oral Infection With *Toxoplasma Gondii* . Eur J Microbiol Immunol (Bp) (2013) 3(3):211–21. doi: 10.1556/EuJMI.3.2013.3.10 PMC3832097 24265941

[57] Fu X Wu B Huang B Zheng H Huang S Gan Y. The Correlation of Tim-3 and IFN-γ Expressions in Mice Infected With *Toxoplasma Gondii* During Gestation. Parasitol Res (2015) 114(1):125–32. doi: 10.1007/s00436-014-4167-3 25270237

[58] Vinay DS Kwon BS. 4-1bb (CD137), an Inducible Costimulatory Receptor, as a Specific Target for Cancer Therapy. BMB Rep (2014) 47(3):122–9. doi: 10.5483/BMBRep.2014.47.3.283 PMC4163883 24499671

[59] Shuford WW Klussman K Tritchler DD Loo DT Chalupny J Siadak AW. 4-1BB Costimulatory Signals Preferentially Induce CD8^+^ T Cell Proliferation and Lead to the Amplification *In Vivo* of Cytotoxic T Cell Responses. J Exp Med (1997) 186(1):47–55. doi: 10.1084/jem.186.1.47 9206996 PMC2198949

[60] Takahashi C Mittler RS Vella AT. Cutting Edge: 4-1BB is a Bona Fide CD8 T Cell Survival Signal. J Immunol (1999) 162(9):5037–40. 10227968

